# Post‐Total Knee Replacement Persistent Bilateral Popliteal Cysts Requiring Surgical Excision: A Literature‐Based Discussion

**DOI:** 10.1155/cro/8740464

**Published:** 2026-05-19

**Authors:** Mineth Heenkende, Yushy Zhou, James D. Stoney

**Affiliations:** ^1^ School of Medicine, Faculty of Health, Deakin University, Melbourne, Australia, deakin.edu.au; ^2^ Department of Surgery, Faculty of Medicine, Dentistry and Health Sciences, The University of Melbourne, Parkville, Australia, unimelb.edu.au; ^3^ Department of Orthopaedics, St Vincent’s Hospital, Australia St Vincent’s Health Service, Fitzroy, Australia, stvincents.ie

## Abstract

We report a patient who developed the unusual complication of persistent symptomatic Baker’s cyst (BC) requiring surgical excision following each of her total knee replacements (TKRs), which were performed 10 years apart. BCs are a common problem and rarely require operative intervention. This patient presented with osteoarthritis and a painful complex BC. A successful left TKR was performed but the BC persisted following the surgery. Conservative management of the BC after the TKR failed to alleviate the symptoms, and surgical excision of the BC was eventually performed. This patient experienced the same problem 10 years earlier in their contralateral (right) knee, which also required excision of a BC after TKR to achieve complete resolution. This case demonstrates that a large complex BC with septations and a thickened wall may not necessarily resolve after TKR and may, on rare occasions, require surgical excision to relieve symptoms.

## 1. Introduction

Popliteal or Baker’s cysts (BCs) are fluid‐filled extensions of a pre‐existing bursa in the popliteal fossa, most commonly the gastrocnemio‐semimembranosus bursa or a synovial pouch [1, 2]. The most common precipitating conditions for BC are degenerative diseases of the knee such as osteoarthritis (OA) and inflammatory arthritis [3–5]. BCs can result from anatomical changes associated with arthritis, such as synovitis, osteophyte formation and joint deformity, but can also be related to increased synovial fluid production due to inflammatory reactions [6, 7].

BCs are common in patients with OA and are therefore often seen as a pre‐existing condition in patients presenting for TKR. BCs much less commonly arise as a new condition after TKR. BCs that present following TKR are likely to be pre‐existent cysts, which fail to resolve following the procedure [6].

The typical adult presentation consists of vague popliteal pain and/or tightness caused by a small BC, which does not affect range of motion [1]. Larger BCs are associated with impaired range of motion secondary to pain and present with Foucher’s sign—a palpable popliteal mass firm in knee extension and soft in flexion.

The commonest complication of a BC is rupture or leakage of the cyst, which can mimic a DVT, with 2%–6% of patients suspected of DVT having a symptomatic BC as the cause [1]. Rarer complications are compression of nearby neurovascular structures and intra‐cystic haemorrhage [1, 5, 8]. Occasionally, BCs can present with atypical findings on ultrasound such as septations, thickened walls and intra‐cystic blood clots [9]. Tsang et al. documented that these atypical findings were reported in 65% of BCs who presented with knee pain [9].

This case report describes a patient who underwent bilateral TKR 10 years apart, with symptomatic BCs that did not resolve post‐TKR, requiring surgical excision for relief. The failure of both the TKR and subsequent conservative management to resolve the BC in both knees is a rare occurrence and, on this patient, surgical excision proved to be an effective management strategy to relive the pain and discomfort from the BC and to prevent recurrence. To our knowledge, this is the first report of bilateral persistent BCs requiring surgical excision following TKR performed a decade apart.

## 2. Case Presentation

A 72‐year‐old female presented to an orthopaedic surgeon with an 18‐month history of left knee pain, as well as a swelling in the popliteal fossa. This was consistent with chronic OA and a concurrent BC. She described a sensation of fullness and tenderness to palpation in the popliteal fossa, indicating the presence of a large, painful mass that prevented comfortable flexion. The knee pain was typical of OA, with a progressive chronology. The pain was worse in the mornings, and it was beginning to impede the patient’s ability to walk long distances. Otherwise, she was fit and well, with no regular medications and independent in all activities of daily living.

Approximately 10 years earlier, the patient presented to another orthopaedic surgeon with similar symptoms in her right knee (OA and a large BC). She underwent a right TKR that was complicated by a persistent and painful right BC that required surgical excision 3 months after her TKR. This resolved the pain and discomfort in her right knee and there were no additional concerns.

On examination of her left knee, a firm ‘mandarin’‐sized swelling was noted in the popliteal fossa. She was able to flex her knee from 0° to 130°. She had a varus alignment. She had medial joint line tenderness. The knee was stable. Hip examination was normal. She was neurovascularly intact.

**Figure 1:**
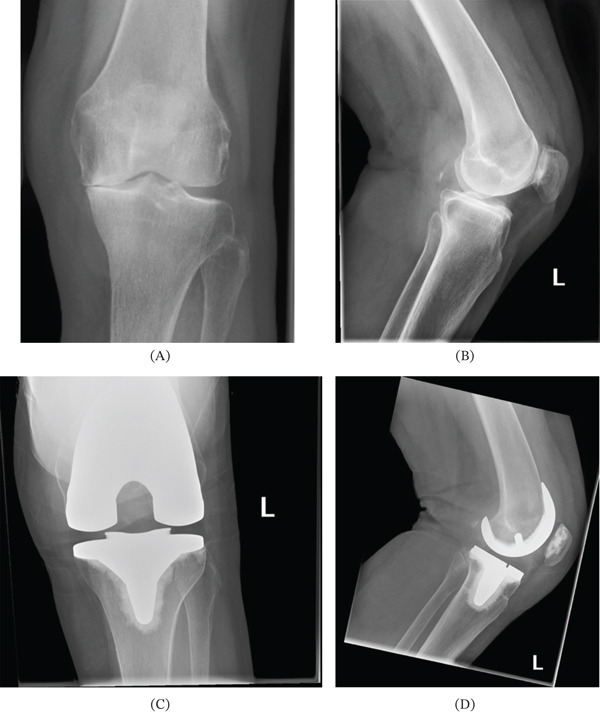
Plain radiographs of the left knee. Anteroposterior (A) and lateral (B) views pre‐TKR show Grade 4 medial compartment osteoarthritis and Grade 3 changes in the lateral compartment. Rosenburg (C) and lateral (D) views post‐TKR show a fully cemented fixed bearing primary TKR in‐situ.

X‐rays demonstrated Kellgren–Lawrence Grade 4 OA in her left knee medial compartment, with Grade 3 changes in her lateral compartment (Figure 1). On long‐leg alignment views, the patient had 4 degrees of varus in her left knee.

The patient had persistent pain and swelling despite 12 months of non‐operative treatment. She was therefore scheduled for a left TKR. The prosthesis was a fixed bearing, fully cemented, primary TKR, performed with navigation assistance (Figure 1). This was an uncomplicated procedure. Prior to the procedure, it was discussed with the patient the possibility of the BC persisting as it did with her right TKR, although this was thought to be unlikely to occur a second time.

**Figure 2:**
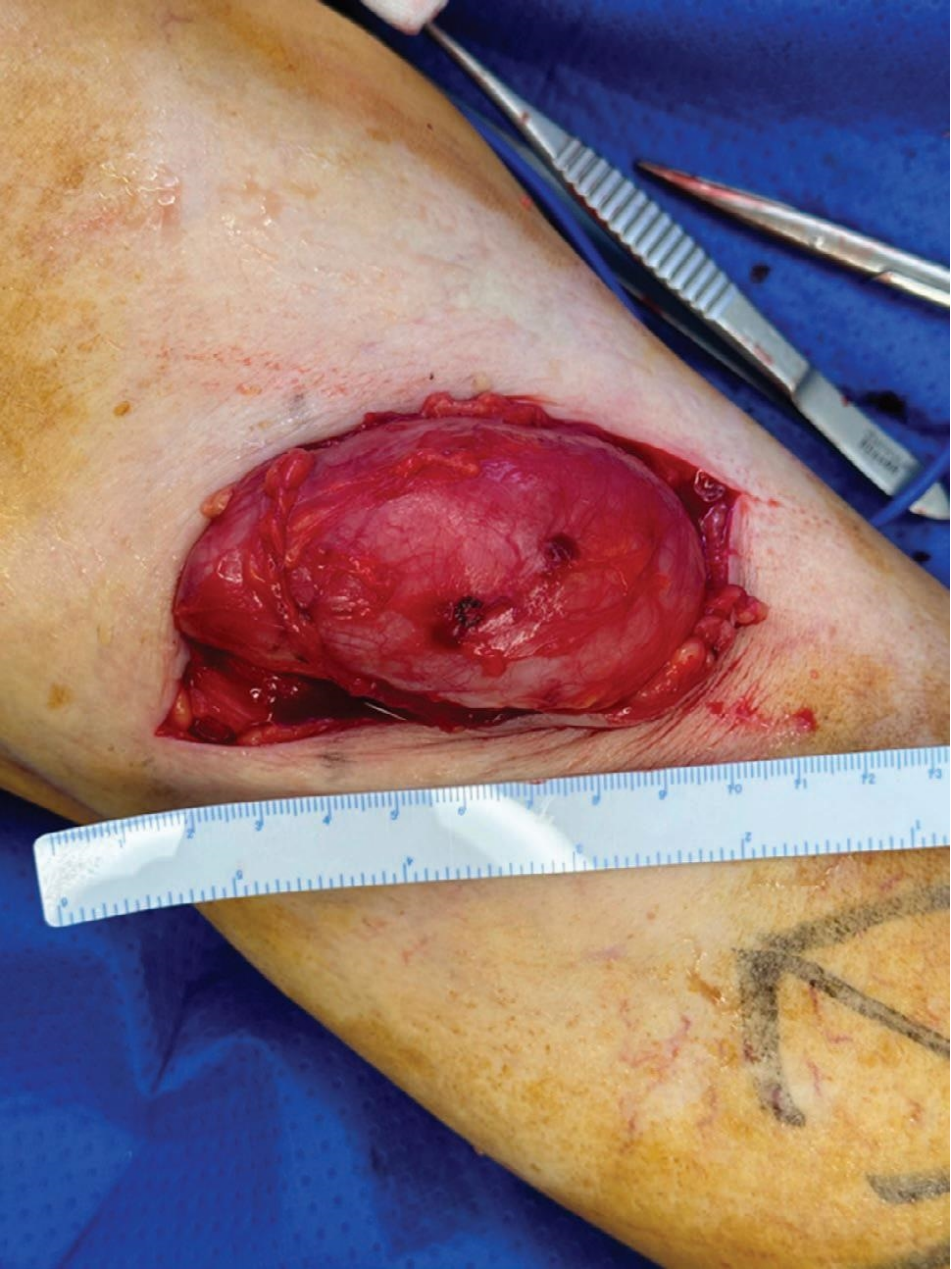
Exposed left knee Baker’s cyst measuring 85 × 60 × 20 mm with wall thickness up to 8 mm.

At the 6‐week follow‐up appointment, the medial compartment knee OA symptoms were improved; however, the patient had difficulty flexing the knee because of mass effect and pain from the BC. An ultrasound‐guided aspirate was performed. A multiloculated cyst with numerous avascular internal septations and no internal vascularity, measuring 10 × 4 × 6 cm was identified, from which 50 mL of clear straw‐coloured fluid was aspirated. Following the aspirate, there was no improvement in the patient’s symptoms and the BC quickly reaccumulated. At 3 months after her left TKR, the decision was made to surgically excise the BC using a posterior approach to the knee in which the neck of the cyst was sutured to prevent recurrence (Figure 2). This successfully relieved the patient of their symptoms.

At 18 months after her left TKR, the patient had no significant left knee pain, no palpable or painful BC remaining and was happy with the overall result of the surgery.

## 3. Discussion

This case report highlights the rare presentation of a 72‐year‐old female with bilateral symptomatic BCs that persisted after TKR performed 10 years apart. The BC required surgical excision on each occasion.

Standard management of BCs include conservative measures such as resting, aspiration, steroid injection and treatment of underlying degenerative changes, prior to considering open cyst excision as a last line therapy [5]. The patient’s experience on both occasions highlights that surgical excision is an effective management strategy for those with persistent, large, chronic BCs that fail to respond to conservative management.

When reviewing the literature, previous reported prevalence of BC after TKR was low at 0.6% [6]. One of the strongest predictors of symptomatic BC after TKR was BC before TKR [10]. In a case series of patients with pre‐existing BC before undergoing TKR, 85% of patients reported persistent BC 1 year after TKR [10]. Despite the high rates of BC persistence even after TKR, only one‐third of patients experienced popliteal symptoms, defined as pain, limited range of motion or inability to perform activities of daily living because of the BC, at 1 year after TKR. When projecting the follow‐up to 5 years after TKR, this proportion of patients with symptoms from their BC increased to approximately one‐half, highlighting the importance of timing of management strategies [10].

Conservative measures have been shown to successfully alleviate symptoms for most patients. Tofte et al. described a small case series of nine patients who had resolution of their BC after TKR [6]. Two of these patients underwent aspiration and steroid injection, three underwent aspiration alone, and a patient each who underwent steroid injection without aspiration, and surgical excision and synovectomy, respectively. In the same case series, the BC in one patient resolved without specific therapeutic management [6].

In another case series, Saylik et al. described 103 knees with symptomatic BC > 3 cm in diameter treated using posterior open cystectomy with valve and posterior capsule repair, including arthroscopic treatment of intra‐articular lesions when required. All patients underwent 6 months of conservative management prior to surgical intervention. At 1 year follow‐up, nine patients reported pain and discomfort with cyst recurrence in two patients for an incidence rate of 2% [11].

Histopathology of either BC were not performed, the details from which may have provided insight as to why the BC persisted in this particular case. Similarly, the exact technique used in the surgical excision of the right knee BC was not able to be found within the available medical records. The lack of this information, along with further investigations with advanced imaging is a limitation of this study, which prevents delineation of a cause for the recurrence of the BC such as biomechanics, valve anatomy or synovitis.

## 4. Conclusion

This case demonstrates a rare occurrence in which a patient had bilateral symptomatic BCs that persisted following TKR, and conservative treatment. Persistent BCs after TKR, although rare, can substantially impact the patient’s quality of life through pain and loss of function. If conservative management fails, surgical excision should be considered as an avenue to achieve resolution of the BC.

## Author Contributions

Mineth Heenkende wrote the manuscript. Yushy Zhou and James D. Stoney provided all relevant clinical data.

## Funding

No funding was received for this manuscript.

## Disclosure

All authors have reviewed the final manuscript.

## Ethics Statement

Ethical approval was not sought for this case report because anonymised patient information was used. Written informed consent was obtained for anonymised patient information to be published in this article.

## Conflicts of Interest

The authors declare no conflicts of interest.

## Data Availability

Data available on request due to privacy/ethical restrictions.
